# A Sustainable Wholesome Foodstuff; Health Effects and Potential Dietotherapy Applications of Yacon

**DOI:** 10.3390/nu11112632

**Published:** 2019-11-03

**Authors:** Mary R. Yan, Robert Welch, Elaine C. Rush, Xuesong Xiang, Xin Wang

**Affiliations:** 1 AUT Food Network, Auckland University of Technology, Auckland 1010, New Zealand; erush@aut.ac.nz; 2 Community and Social Sciences, Unitec Institute of Technology, Auckland 1025, New Zealand; 3 Yacon New Zealand Ltd., Auckland 1051, New Zealand; raswelch@outlook.com; 4 National Institute of Nutrition and Health, China CDC, Beijing 100050, China; xiangxs@ninh.chinacdc.cn; 5 Plant Protection and Microbiology, Zhejiang Academy of Agricultural Sciences, Hangzhou 310021, China; xxww101@sina.com

**Keywords:** yacon, public health, chronic diseases, prebiotic, fructooligosaccharides, inulin, phenolic compounds

## Abstract

A sustainable food supply is an ever-growing public and planetary health concern influenced by food culture, food practices, and dietary patterns. Globally, the consumption of plant foods that offer physiological and biochemical benefits is increasing. In recent years, products made from yacon (*Smallanthus sonchifolius*) tubers and leaves, e.g., in the form of syrup, powder, and herbal tea, have steadily emerged with scientific evidence to validate their possible health claims. Yacon was introduced to New Zealand in 1966, and its products can now be produced on a commercial scale. This paper reviews literature published mainly in the last 10 years concerning the health-related properties of yacon as a wholesome foodstuff and its bioactive components, e.g., fructooligosaccharides. Literature was sourced from Web of Science, PubMed, EBSCO Health, and Google Scholar up to June 2019. The potential markets for yacon in the field of food technology and new dietotherapy applications are discussed. Furthermore, the unique features of New Zealand-produced yacon syrup are introduced as a case study. The paper explores the scientific foundation in response to the growing public interest in why and how to use yacon.

## 1. Introduction

A sustainable food supply is an ever-growing public and planetary health concern influenced by food culture, food practices, and dietary patterns. Globally, the consumption of plant foods that offer physiological and biochemical benefits is increasing [1,2]. In recent years, products made from yacon (*Smallanthus sonchifolius*) tubers and leaves, such as syrup, powder, and herbal tea, have steadily emerged with scientific evidence to validate their applications. Yacon is perceived as a functional food because it contains biologically active components that may provide physiological benefits beyond basic nutritional functions to reduce the risk of chronic diseases [3,4]. Yacon, originally from South America, is now consumed in some countries such as Japan, South Korea, and China, but it is relatively new to the global market [5,6]. Its health-related properties are closely associated with its abundant bioactive components [7,8].

In vivo studies in animal models have shown yacon’s biological effects on glycemic control, as well as its reductions of plasma cholesterol and low density lipoprotein (LDL) [9,10,11,12]. Clinical evidence from human studies is currently limited. Some studies have demonstrated that the consumption of yacon has positive health effects including anti-cancer [13], anti-inflammatory and antioxidant activity related to its phenolic compounds [14], immunity improvement in preschool children [15], diabetes management in the elderly [16,17], weight management, and obesity prevention in overweight adults [17,18,19,20], all of which have been reported to be associated with the content of fructooligosaccharides (FOS) and inulins. In addition, yacon has potential markets in the development of new food products and new dietotherapy applications [4,5,21].

This paper reviews literature published mainly in the last 10 years that concerns the nutritional and chemical composition of yacon, the health-related properties of yacon as a wholesome foodstuff, and the bioactive components of yacon. Literature was sourced from Web of Science, PubMed, EBSCO Health, and Google Scholar up to June 2019. The potential markets for yacon in the field of food technology and new dietotherapy applications are discussed. Further, the unique features of New Zealand-produced yacon syrup are introduced as a case study. The paper explores the scientific foundation in response to the growing public interest in why and how to use yacon.

## 2. Yacon, and Nutritional and Chemical Composition

Yacon is a plant native to South America, where it is consumed as food and is used in folk medicine for treating diabetes, constipation, and other diseases [7,22]. The plant grows well in warm, low frost areas and, in most conditions, does not require pesticides for protection from fungi and insects.

**Figure 1:**
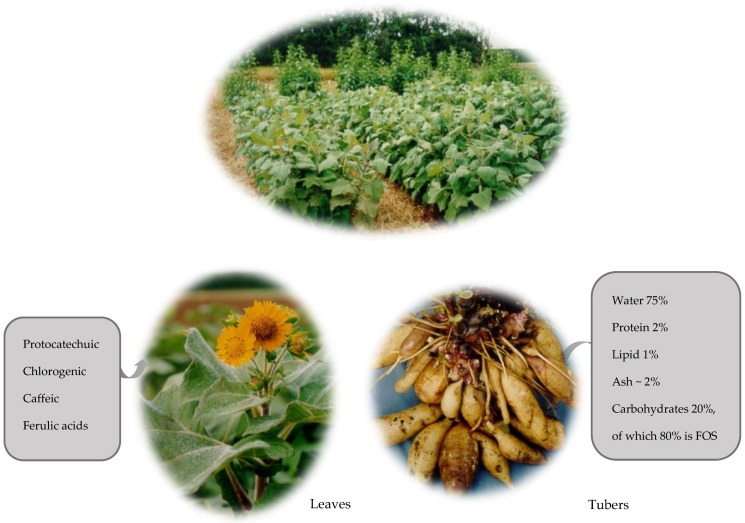
Yacon plants grown in New Zealand, annotated with nutritional and chemical composition of yacon leaf and tuber (photos: Crop and Food Research New Zealand).

Yacon is a perennial herbaceous plant that has sweet-tasting-tuberous roots (Figure 1). The roots are often referred to as tubers, have a tan-colored thin skin, and have crisp flesh. The main substances in fresh yacon roots are: water (>70%), carbohydrates (20%, of which 80% are FOS and inulin), protein (2%), lipid (1%), and ash (2%) [7,8]. The content of carbohydrates in the dry matter of yacon roots is approximately 94% [7]. Apart from free sugars, e.g., glucose, fructose, and sucrose, yacon roots store carbohydrates in the form of fructans, unlike most plant roots in the human diet that store carbohydrates in the form of starch [17,21,23]. A fructan is a polymer of fructose molecules. Fructans with a short-chain are known as FOS (the degree of polymerization (DP) < 9), and long-chain linear fructans are inulins (DP up to 60). Both FOS and inulin are *β*-D-fructafuranoses joined by a *β*-(2,1) bond [24].

Both the leaves and tubers of yacon contain significant quantities of bioactive compounds. The leaves of yacon contain protocatechuic, chlorogenic, caffeic, and ferulic acids (Figure 1), which give antidiabetic and antioxidant properties to infusions (herbal teas) made from the leaves [25,26,27]. The roots of yacon contain antioxidants, fructose, glucose, sucrose, and *β*-(2,1) fructooligosaccharides (inulin-type oligofructans) [6,7,8]. Inulin-type oligofructans are fermented by beneficial species of gut bacteria and may thus be prospective prebiotics [28]. In addition, phenolic acids occur in both the leaves and roots of yacon. Some compounds have been identified as caffeic acid and its derivatives, chlorogenic acid and L-tryptophan [29]. Moreover, yacon roots have a high amount of phenolic compounds (about 200 mg in 100 g of edible fresh matter) compared to other plant roots and tubers [21].

The chemical composition of yacon leaves and tubers may vary depending on factors including planting location, growing season, and harvest time. The FOS content in New Zealand-grown yacon tubers has been shown to vary from 58% to 78% of the total carbohydrates [30]. The ratio of FOS to free sugars depends on the stage of development of the crop, as well as the time and temperature of post-harvest storage.

## 3. Health Effects (Usually Benefits) of Yacon and Its Bioactive Components

Human studies on yacon are currently limited. Most of the in vivo studies have been conducted in animal models. However, studies on individual components such as FOS, inulin, and phenolic compounds could reflect the health effects or benefits of yacon to humans. The health effects of either yacon as a whole or each functional component of yacon are now reviewed in detail.

### 3.1. Health Effects of Fructooligosaccharides (FOS)

Fructooligosaccharides are short-chain oligosaccharide fructans (DP < 9) that occur naturally in the cell vacuoles of plant leaves, stems, and roots. FOS can be extracted from plants, fruits, vegetables, and some grains and cereals such as wheat and barley. The content of FOS and inulin-type fructans is up to 70% of the dry matter of yacon roots. The highest concentration of FOS of cultured plants has been found in yacon [21,23,31] in the order of 16% fresh weight.

As soluble fibers, FOS are commonly used as a low-calorie alternative sweetener and is now increasingly popular for its prebiotic effects [22,32]. This is because amylases cannot hydrolyze *β*-(2,1) bonds, and FOS are resistant to enzymatic hydrolysis by salivary and intestinal digestive enzymes, thus passing through the upper gastrointestinal tract without being metabolized before it is fermented by anaerobic bacteria in the colon to terminal products—short chain fatty acids (SCFA) [33,34]. The human intestinal microflora are composed of more than 400 species, and the colon is the region with the highest microbial population. FOS have an indirect influence on immunity through SCFA production. Research in animal models has suggested that SCFA production by FOS fermentation in the colon can increase local immune response, reduce colon pH, and, therefore, suppress inflammation and in the longer term development of colorectal cancer [35,36].

**Figure 2:**
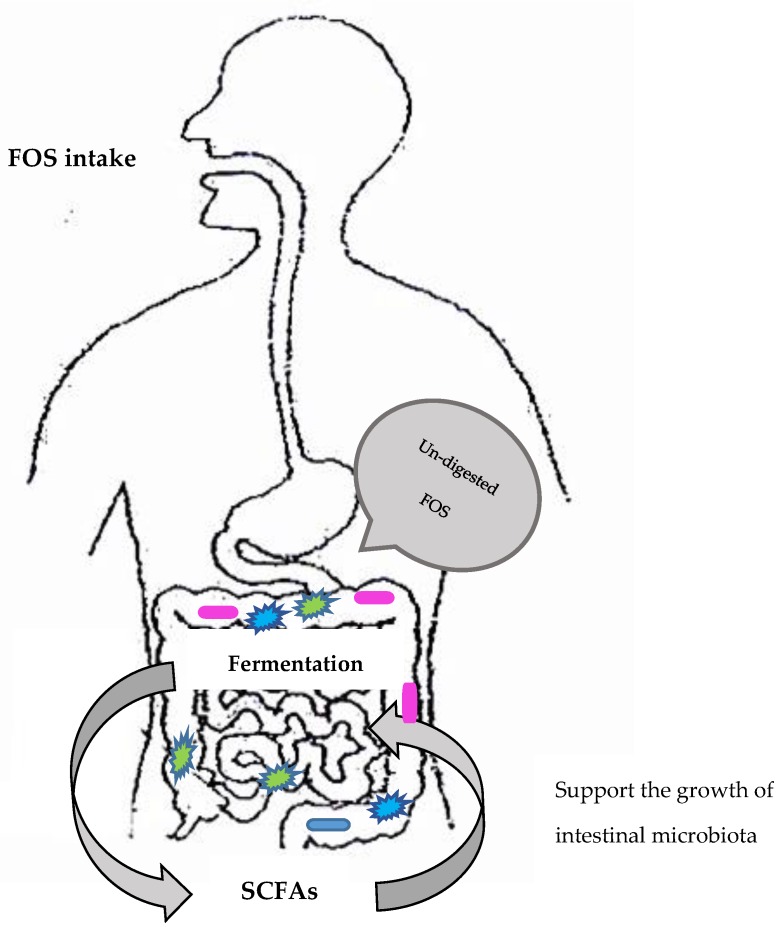
Fructooligosaccharides (FOS) work in the gastrointestinal tract as prebiotics; SCFAs: short chain fatty acids.

At the genus level, both FOS and SCFA support the growth of beneficial bacteria such as *Bifidobacteria* spp. and *Lactobacillus* spp. [7,37,38,39] (Figure 2). *Bifidobacteria* constitute 25%–30% of the total population of the gut bacteria, while *Lactobacillus* constitute less than 1% of the gut bacterial population. Probiotic strains of *Lactobacilli* and *Bifidobacteria* potentially alter gut microbiology. Their optimization in the intestinal microflora can ease constipation, improve serum lipids in hyperlipidemia, and suppress the production of intestinal putrefactive substances in the digestive tract.

In a detailed mouse model, Delgado et al. [40,41] reported that the consumption of fructans increased immune system efficiency. The daily intake of FOS for 30 days has been shown to lead to an improvement in anti-inflammatory state in phagocytic cells and mucosal immunity associated with reduced risks for autoimmune and metabolic diseases. Velez et el. [42] found that the oral administration of yacon root flour in mice regulated intestinal microbiota balance and had immunomodulatory effects without inflammatory responses. Moreover, they demonstrated that yacon flour could be useful in preventing infection caused by *Salmonella typhimurium*. A human study in preschool children (*n* = 59, age of 2–5 years) demonstrated improved intestinal immune responses shown by secretory immunoglobulin A (IgA) concentrations after the daily consumption of 0.14 g of FOS per kilogram of body weight for 18 weeks [15]; there was no effect on the nutritional status of iron and zinc.

In other studies, FOS supplementation has been shown to improve growth performance [43], mineral absorption [44], and bowel function [45].

Due to its non-digestible property, FOS have a low glycemic impact. A randomized, double-blind trial in obese adults (body mass index (BMI) 25–30 kg m^-2^) reported that the daily intake of 20 g of FOS (*n* = 40) for three months resulted in a significant reduction of atherogenesis and body weight, compared with placebo control (*n* = 32) [18].

FOS are recognized as generally safe by the Food and Drug Administration (FDA) and verified by clinical studies [46,47]. However, the side effects of FOS consumption including digestive upset and abdominal stress should be taken into account when used as dietary supplements or food ingredients, in particular in baby formula [48].

### 3.2. Health Effects of Inulin

Inulins are fructan-type polysaccharides (DP up to 60) that occur in many plant roots, fruits, vegetables, and some grains and cereals. Inulin and FOS, both fructans, are the main constituents in the dry matter of yacon roots [4,8]. The favorable characteristics that apply to FOS are very likely to apply to inulins.

Inulin is soluble in water and therefore classed as soluble fiber [49]. Because of the *β*-(2, 1) linkages, inulin is not digested by enzymes in the upper gastrointestinal tract but is fermented in the colon, contributing to its reduced calorie value and prebiotic properties. Inulin is commonly used in the food industry as a low-calorie sweetener; a texture modifier in dairy products such as yogurt, cheese, milk drinks [49,50]; and as dietary fiber and prebiotics used in functional foods [51,52].

Similar to FOS, the functional effects of inulins include promoting digestive health as prebiotics and contributing to the production of colon butyrate, which is associated with a reduced risk of colon cancer and breast cancer [21,53]. Moreover, due to its non-digestible feature, inulin has a favorable impact on blood glucose concentration when consumed with other foods. Inulin supplementation has been positively associated with a reduced fasting glucose and fasting insulin concentration in type 2 diabetes patients [54].

Inulins are generally recognized as safe [46,47]. Their side effects may include intestinal discomfort and allergic reactions [55].

### 3.3. Health Effects of Phenolic Compounds

Phenolic compounds are a dominant class of secondary metabolites in plants which comprise hydroxy groups bonded to an aromatic hydrocarbon groups (benzene rings) and range from simple to high polymeric compounds [56,57]. Compared to other plant roots and tubers, yacon roots have a high concentration of phenolic compounds, about 200 mg in 100 g of edible fresh matter [21].

Recently, phenolic compounds have been of growing interest to health professionals and the food industry for their potential health benefits—antioxidant properties, in particular. Phenolic compounds have antioxidant capacities that are related to the hydroxyl groups and the conjugated double bonds of the benzene ring [58], which together act as quenching agents of deleterious free radicals. Epidemiological studies have suggested the potential health effects of phenolic compounds in the prevention of many chronic diseases such as diabetes, cancers, and cardiovascular diseases [59,60], all potentially arising from their antioxidants, anti-inflammatory [61,62], and anti-carcinogenic functional properties [63,64].

Because of the anti-inflammatory effect associated with antioxidant activity, polyphenols have been proposed to be useful for the development of future antioxidant therapeutics and anti-inflammatory drugs [62].

### 3.4. Health Effects of Yacon Leaf and Tuber

Though it is relatively new to the global market due to its lack of availability and low popularity, yacon has been considered as a functional food with multiple physiological properties related to its bioactive compounds [4,5] in both its leaves and tubers; these properties include those of anti-cancer, antioxidative, antimicrobial, antidiabetic, anti-obesity and weight management. These properties can almost certainly be ascribed to FOS, inulin and phenolics, and this is the theme of the studies described now.

Yacon leaves have been traditionally used in the Andes to treat people suffering from diabetes and digestive diseases. In experimental trials, dos Santos et al. [12,65] reported a significant reduction of glycemia, an increase in insulin concentration, and a decrease in serum triacylglycerol concentration in streptozotocin-induced diabetic rats after the oral administration of yacon leaf extract for 30 days. Valentova et al. [66] and other studies [67,68,69,70] reported the anti-hyperglycemic effects of yacon leaf extract in animal models.

Yacon leaves are pest-resistant and antimicrobial, and these properties are associated with the content of monoterpenes, sesquiterpenes, and diterpenes [25,71]. In in vitro studies, sesquiterpene lactones from yacon leaves have been shown to strongly inhibit cancer cell deformation and proliferation [13,72,73,74]. The extracts of dried leaves have shown antioxidant function related to their phenolic content. Valentova et al. [26] proposed that yacon leaves can be used as a potential remedy in the prevention of chronic diseases caused by free radicals, e.g., arteriosclerosis. Moreover, Oliveira et al. [75] reported the topical anti-inflammatory activity of yacon leaf extracts. The extracts were proven to have in vivo anti-edematogenic activity and could be potential anti-inflammatory agents.

Yacon roots are rich in phenolic compounds and are therefore a potential novel source of antioxidants [14,39,76]. Moreover, because of its high content of inulin and FOS (up to 70% of the dry matter of the yacon root), yacon has shown important prebiotic characteristics.

Yacon roots possess anti-cancer, antioxidative, and antimicrobial properties [7,77,78,79,80,81,82]. An ethanol extract of yacon, which is rich in phenolics, inhibited the cell proliferation and migration of C6 glioma cells stimulated with fetal bovine serum [81]. The oral administration of the aqueous extract of yacon roots and *Lactobacillus acidophilus* had protective effects against colon carcinogenesis on the early phases of tumor development in rats [77,83].

Studies in preschool children (*n* = 59, age of 2–5 years) have demonstrated improved intestinal immune responses shown by secretory IgA concentrations after the daily consumption of yacon root flour (0.14 g of FOS per kilogram of body weight) for 18 weeks [15], but no effect on the nutritional status of iron and zinc was demonstrated. Some studies have reported improved mineral absorption in animal models after the consumption of yacon flour containing fructan [10,84,85].

The antidiabetic effects of yacon roots have been proven in animal models and human studies. An improvement of biochemical parameters in type 1 diabetic rats was observed after oral treatment by the aqueous extract of yacon roots [80]. The hypoglycemic effects of yacon tuber extracts and their phenolic constituent, chlorogenic acid, have been demonstrated in diabetic rats [11]. Habib et al. [78,79] reported that the administration of FOS-rich yacon flour to diabetic rats for 90 days led to a significant decrease in fasting plasma triacylglycerol and very low density lipoprotein concentrations but a slight increase in fasting plasma insulin concentrations [78], as well as a significant decrease in malondialdehyde levels in both liver and kidney [79]. Malondialdehyde is a marker of deleterious fat oxidation. Furthermore, a yacon-enriched diet has been shown to improve insulin resistance in insulin resistant rats that could favor blood glucose control [41]. In a controlled trial with elderly subjects (aged over 60 years), the daily consumption of 18 g of freeze-dried yacon powder containing 7.4 g of FOS for nine weeks resulted in a decrease in serum glucose, but no reduction was observed in serum lipids [16].

The effects of yacon on weight management and obesity prevention are mainly related to the high content of the non-digestible FOS and phenolic compounds. Feeding rats with yacon flour for 12 weeks has demonstrated an improvement of visceral adiposity and metabolic parameters [86]. The administration of an aqueous extract of yacon root containing 4.3% of fructans (1 mL of the extract per kilogram of body weight) for seven weeks has been shown to improve lipid profiles by a reduced triacylglycerol concentration and an increased high-density lipoprotein cholesterol concentration in diabetic rats [87]. In human studies, the daily consumption of a breakfast drink containing 25 g of yacon flour for six weeks by overweight adults (BMI 30 ± 2.4 kg m^−2^, body fat 40 ± 6.7%) resulted in reduction in body weight, body fat, waist circumference, and sagittal abdominal diameter [19].

In terms of promoting gastrointestinal health, Utami et al. [88] demonstrated that compared with commercial FOS, yacon tuber (containing same amount of FOS) consumption promoted the growth of *Bifidobacteria* and *Lactobacillus* in the rat cecum, resulting in a greater concentration of SCFAs. They concluded that the yacon tuber has more favorable effects in colonic health maintenance than that of other FOS sources. It has been proposed that the yacon tuber also contains resistant starch and polyphenols, both of which contribute to the difference in gut environment.

In behavioral despair animal models (mice), antidepressant-like effects have been observed after inulin-type oligosaccharides extracted from yacon roots were administered; the results suggested that yacon consumption has the potential to treat patients with depression [89].

The possible adverse effects of yacon have been investigated in animal models and human studies. Genta et al. [90] reported that no toxicity and adverse side effects were observed in rats after four months of the regular consumption of yacon root flour that provided 340 mg of FOS per kilogram of body weight (this dose is likely the upper limit of human tolerance in long-term trials); in a human study, there were no ill effects after 120 days of the supplementation of yacon syrup that provided 0.14 g of FOS per kilogram of body weight [17]. However, there were side effects reported in two studies. One study reported the development of renal lesions in rats after 30 days of oral treatment with the aqueous yacon leaf extract, where terpenoids were proposed to be the main toxic compounds in yacon leaves [91]. Another study reported the development of anaphylactic reactions in a 55-year-old woman after the ingestion of yacon root [92]. This appears to be an isolated response, but further studies are needed to investigate the safety of yacon used as dietary supplements.

### 3.5. Others

Human and animal studies have demonstrated the health benefits of the consumption of yacon in relation to health promotion and chronic diseases prevention. The in vitro and in vivo studies on yacon in human or animal models are summarized in Table 1.

## 4. Potential Dietotherapy Applications: How to Use Yacon

The global market value for prebiotics is currently worth more than US$ 3 billion. The concept is well-known in Japan, Taiwan and South Korea, and it is becoming known in North America and Western Europe. Yacon has a large potential in the application of food technology and in the prevention of chronic diseases where, for instance, it is a potential novel source of prebiotics. Yacon tubers can be consumed raw, cooked, or in the form of jam, syrup, vinegar, flour, chips (dried slices), and juice as a novel food. The flesh is crisp like a nashi pear or water chestnut if consumed raw. In Japan, yacon roots are processed into bakery products, fermented beverages, freeze-dried powder, and other products [4,5,23]. The market position of FOS from yacon is natural and unprocessed. As a food with multiple functions, yacon has potential dietotherapy applications that are associated with its physiological and biochemical properties.

### 4.1. Yacon Tea

Yacon leaves are often used for herbal tea. Medicinal teas made from yacon leaves have been shown to present hypoglycemic and hypocholesterolemic activities [26,66].

In Japan, the leaves of yacon are processed and marketed as an herbal tea in local markets. Sugahara et al. [94] examined the effective antioxidative effects and suggested that yacon herbal tea was a good natural source of antioxidants against free radicals.

### 4.2. Yacon Syrup

Yacon syrup is a novel product obtained from concentrating the juice of yacon tubers. Yacon syrup is comprised of FOS, inulin, and a small amount of free sugars. It is used as a popular sugar substitute due to its sweet flavor and low calorific impact [98].

To date, human trials have been very limited. There have been two studies reporting the satiety-enhancing and anti-obesity effects in humans. The daily consumption of yacon syrup has been shown to increase satiety sensation [17,20] and to result in a significant decrease in body weight over a 120-day period [17]. Yacon syrup could be well-positioned as a nutraceutical product because of its naturally high FOS content [17]. A daily intake of 20 g of FOS or less in humans is generally considered safe [90].

### 4.3. Yacon Powder

The commercial yacon powder products are available in health food and organic stores in producer countries and Europe. The suggested use of yacon powder is as a dietary supplement.

### 4.4. Yacon Capsules

Yacon root extract is packed in the form of quick-release gelatine capsules for convenience. Human studies on yacon capsules could not be found.

### 4.5. Yacon Used in Infant Formula

Yacon has a potential application in infant formulas due to its high content of non-digestible carbohydrates [99,100], while considering that FOS are often included in formula.

### 4.6. Other Uses of Yacon

Other applications of yacon include its use as a natural source to treat depression [89], its use in low-calorie bakery products (e.g., cake) as a partial substitute for flour related to its content of FOS and inulin [101], and its use in beverages [102], in yoghurt [20] or in juice mixtures in Korea.

Yacon has a history of safe use as foodstuff and a source of prebiotics [23]. FOS (the major bioactive component of yacon) have GRAS (generally regards as safe) status recognized by the FDA and its Japanese equivalent. Though the legal status of using yacon leaves as herbal tea could not be sourced, the safe use of yacon leaves for their hypoglycemic effect was demonstrated by Barcellona et al. [103]. There are no known regulatory issues in South Korea and Taiwan. The Food Safety Authorities of Australia, New Zealand, and European have approved the sale of yacon products in their markets.

## 5. Yacon New Zealand: A Case Study

**Figure 3:**
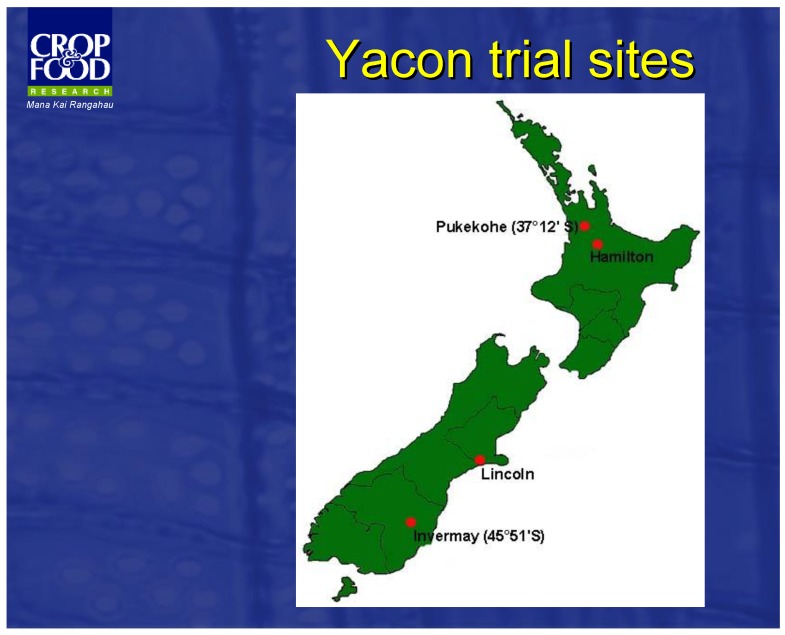
Yacon plants were brought to New Zealand by Crop and Food Crown Research, and trials were carried out in small plots in different regions on the basic agronomy of the crop to optimize growing and harvesting conditions.

The yacon plant was brought to New Zealand in 1966. It was introduced to Japan in 1985, and from there, it was introduced to Korea, China, Indonesia, the Philippines and Brazil. Since 1995, the Crop and Food Crown Research Institute has investigated the basic agronomy of yacon, and trials were carried out in small plots in eight regions to optimize growing and harvesting conditions (Figure 3). With its great volcanic soil, free draining, strong ultraviolet radiation, and mild climate, New Zealand has the optimized conditions for growing high-FOS producing yacon. Yacon production is benefiting from a tradition of high quality crop and horticulture production in New Zealand. In 2000, Yacon New Zealand Ltd. purchased propagative material and the right to exclusive development. The crop is currently grown and its products can be produced on a commercial scale. The composition of the tubers is monitored during development, and they are harvested at the time of peak FOS content.

Khajehei et al. [14] reported that New Zealand yacon has the lowest sugar content of all yacon varieties. Yacon New Zealand Ltd. produces yacon juice and further concentrated yacon syrup. Yacon juice has been sold to Korea and used as an ingredient in yoghurts or juice mixtures. The commercial product yacon syrup (NZFOS+) contains the purest natural prebiotic FOS from New Zealand-grown yacon. The product was tested for its glycemic index (GI) using the international standard methods ISO 26642:2010(E) [104] at the University of Sydney. Ten healthy subjects were recruited for the test. A standard 25 g glucose drink was used as a reference food. The results revealed that New Zealand-produced yacon syrup was low in its GI (GI = 40 ± 4). Ethical approval was obtained from the Human Research Ethics Committee of the University of Sydney.

Further work including in vitro fermentation investigation and clinical trials on yacon syrup (NZFOS+) will be conducted at Plant Protection and Microbiology, Zhejiang Academy of Agricultural Sciences in China.

## 6. Conclusions

In vitro and in vivo studies on yacon have shown the potential health benefits and dietotherapy applications of yacon associated with the maintenance of health and wellbeing, as well as the prevention of chronic diseases. Studies have also demonstrated that yacon is a safe food supplement without significant adverse side effects. Further investigations are needed for human studies and new applications and uses of yacon. In terms of yacon products such as yacon syrup and tea, these options require labelling, branding, marketing and distributing to appropriate markets such as Japan, Taiwan, South Korea, North America, and Europe where these concepts are accepted and the sale of yacon products has been approved.

## Figures and Tables

**Table 1:** Summary of studies on yacon (*Smallanthus sonchifolius*) in vitro and in vivo in human or animal models.

Author(s)	Year	Stated Yacon Source	Component(s) Under Study	Study Design		Physiological Effect(s)
				Method	In Vivo (Human Study or Animal Model)	
Cocato et al. [10]	2019	Yacon root flour	FOS*		Rats (Wistar)	Improved mineral absorption
Machado et al. [19]	2019	Yacon root flour	FOS		Human (BMI 30 ± 2.4 kg m^-2^)	Improved body composition
dos Santos et al. [65]	2018	Yacon leaf extract			Rats (Wistar)	Significant reduction in glycemia
Gomes da Silva et al. [20]	2017	Yacon syrup			Human	Improved satiety effects
dos Santos et al. [12]	2017	Yacon leaf extract			Rats (Wistar, diabetic)	Improved glycemic control
An et al. [89]	2016	Yacon root extract	Inulin		Mice (Kuming); Rats (Sprague Dawley)	Antidepressant-like effects
Baroni et al. [70]	2016	Yacon leaf extract	Phenolics		Rats (Wistar, diabetic)	Improved insulin sensitivity
da Silva et al. [77]	2015	Yacon root extract	FOS		Rats (Wistar)	Anti-cancer (against colon carcinogenesis on the early phases)
de Ford et al. [13]	2015	Yacon leaf extract		In vitro		Anti-cancer
Habib et al. [79]	2015	Yacon root flour			Rats (Wistar, diabetic)	Antidiabetic effects
Jimenez et al. [93]	2015	Yacon root	FOS		Rats (Wistar)	Improved intestinal microflora
Lee et al. [81]	2015	Yacon extract		In vitro		Anti-cancer (inhibited cell proliferation)
Russo et al. [27]	2015	Yacon leaf extract	Phenolics	In vitro		Antioxidant effects
Sousa et al. [82]	2015	Yacon tuber flour	Phenolics	In vitro		Antioxidant effects
Sugahara et al. [94]	2015	Yacon leaf extract	Phenolics	In vitro		Antioxidant effects
Scheid et al. [16]	2014	Yacon root powder	FOS		Human (>60 years old)	Diabetes management, no side effects observed
Vaz-Tostes et al. [15]	2014	Yacon root flour	FOS		Human (ages 2–5 years old)	Improved systemic immunity
Oliverra et al. [75]	2013	Yacon leaf extract		In vitro		Anti-inflammatory
Oliverra et al. [80]	2013	Yacon root extract	Fructan		Rats (induced type 1 diabetic)	Antidiabetic effects
Satoh et al. [95]	2013	Yacon tuber extract			Rats (Zucker fa/fa)	Improved insulin sensitivity
Utami et al. [88]	2013	Yacon tuber powder	FOS		Rats (Sprague Dawley)	Improved intestinal microflora
Velez et al. [42]	2013	Yacon root flour	FOS		Mice (BALB/c)	Regulated intestinal microflora
Campos et al. [39]	2012	Yacon root flour	FOS		Guinea pig	Improved intestinal microflora
Delgado et al. [41]	2012	Yacon root flour	FOS		Mice (BALB/c)	Improved immunity efficiency
de Moura et al. [83]	2012	Yacon root extract	FOS		Rats (Wistar)	Anti-cancer (against colon carcinogenesis on the early phases)
Roselino et al. [87]	2012	Yacon root extract	Fructan		Rats (Wistar)	Improved lipid profiles
Habib et al. [78]	2011	Yacon root flour	FOS		Rats (Wistar)	Antidiabetic and hypolipidemic effects
Lobo et al. [84]	2011	Yacon root flour	Inulin		Rats (Wistar)	Improved mineral absorption
Siriwan et al. [72]	2011	Yacon leaf extract		In vitro		Anti-cancer (inhibited cancer cell proliferation)
Siriwan et al. [73]	2011	Yacon leaf extract		In vitro		Anti-cancer (inhibited cancer cell proliferation)
Bonet et al. [96]	2010	Yacon root flour			Mice (BALB/c)	Improved intestinal microflora (growth of *Bifidobacteria* and *Lactobacilli*)
Genta et al. [68]	2010	Yacon leaf extract	Phenolics		Rats (Wistar)	Hypoglycemic effects
Joung et al. [71]	2010	Yacon leaf extract		In vitro		Antimicrobial effects
Raga et al. [69]	2010	Yacon leaf extract	Phenolics		Mice (albino)	Hypoglycemic effects
Yun et al. [92]	2010	Yacon root ingestion			Human (a 55-years-old woman)	Anaphylaxis^1^
Genta et al. [17]	2009	Yacon syrup			Human (obese with mild dyslipidemia)	Improve insulin-resistance, and satiety effects
Park et al. [11]	2009	Yacon tuber extract			Rats (Sprague Dawley, diabetic)	Improved glycemic control
Baroni et al. [67]	2008	Yacon leaf extract	Phenolics		Rats (Wistar)	Hypoglycemic effects
Geyer et al. [97]	2008	Yacon syrup	FOS		Human	Improved the colonic transit
Lobo et al. [85]	2007	Yacon root flour			Rats (Wistar)	Improved mineral absorption
Genta et al. [90]	2005	Yacon root flour	FOS		Rats (Wistar)	No adverse side effects observed^2^
Valentova et al. [66]	2004	Yacon leaf extract			Rats (diabetic)	Anti-hyperglycemic effects

^*^ FOS: fructooligosaccharides. ^1^ A reported case of adverse side effects. ^2^ Toxicity study.
